# Pain Interference: A Barrier for Daily Living Activities in Older Adults with Multisite Musculoskeletal Pain

**DOI:** 10.1093/geroni/igaa057.1697

**Published:** 2020-12-16

**Authors:** Yael Koren, Suzanne Leveille

**Affiliations:** 1 University of Massachusetts Boston, Dedham, Massachusetts, United States; 2 University of Massachusetts Boston, Boston, Massachusetts, United States

## Abstract

Almost half of older adults experience multisite musculoskeletal pain (MMP) contributing to difficulty in daily activities but little is known about specific domains by which pain interferes in daily living. This study aims to determine domains of pain interference (PI) related to MMP in a cohort of older adults living in the community. The MOBILIZE Boston Study (MBS) is a cohort study of 749 adults aged ≥70y. Musculoskeletal (MSK) pain was assessed using the joint pain questionnaire and grouped as: no pain, single site, and multisite pain. The Brief Pain Inventory PI sub-scale assessed level of interference (0-10 rating) in 7 categories in the previous week including general activity, mood, walking, work, relationships with people, sleep, and enjoyment of life. Interference items were grouped as: none (0 rating), mild (>0, ≤2), moderate ( ≥2, ≤5), and severe (≥5) PI. There was a strong gradient of PI according to pain groups with severe walking interference in 36.5% of those with MMP compared to 3.8% of those with no MSK pain. The least PI was in relationships with others (9.1% of MMP vs 1.1% of no MSK pain). Reports of interference in other domains were intermediate (20-26% of MMP vs 3-4% of no MSK pain). Women and those with Less education reported the most PI in every domain but no differences were observed by age. Greater attention to specific domains of pain interference such as walking could have substantial benefits for reducing the overall impact of MMP among older adults.

